# High-intensity interval training improves bone remodeling, lipid profile, and physical function in multiple sclerosis patients

**DOI:** 10.1038/s41598-024-66448-5

**Published:** 2024-07-13

**Authors:** Alessandra Amato, Patrizia Proia, Anna Alioto, Carlo Rossi, Andrea Pagliaro, Paolo Ragonese, Giuseppe Schirò, Giuseppe Salemi, Rosalia Caldarella, Sonya Vasto, Robert Nowak, Dorota Kostrzewa-Nowak, Giuseppe Musumeci, Sara Baldassano

**Affiliations:** 1 Department of Biomedical and Biotechnological Sciences, Section of Anatomy, Histology and Movement Science, School of Medicine, University of Catania, Via S. Sofia No 97, 95123 Catania, Italy; 2 Sport and Exercise Sciences Research Unit, Department of Psychology, Educational Science and Human Movement, University of Palermo, 90144 Palermo, Italy; 3 Department of Biomedicine, Neuroscience and Advanced Diagnostics, University of Palermo, 90127 Palermo, Italy; 4 Department of Laboratory Medicine, “P. Giaccone” University Hospital, University of Palermo, 90127 Palermo, Italy; 5 Department of Biological Chemical and Pharmaceutical Sciences and Technologies (STEBICEF), University of Palermo, 90128 Palermo, Italy; 6 Institute of Physical Culture Sciences, University of Szczecin, 17C Narutowicza St, 70-240 Szczecin, Poland; 7 Department of Pathology, Pomeranian Medical University in Szczecin, 1 Unii Lubelskiej St, 71-242 Szczecin, Poland; 8 Department of Clinical and Molecular Biochemistry, Pomeranian Medical University in Szczecin, 72 Powstańców Wlkp. Al, 70-111 Szczecin, Poland

**Keywords:** Biochemistry parameters, Bone markers, Muscle physiology, Training, Coordination, Multiple sclerosis, Sport, Adapted physical activity, Biochemistry, Chemical biology, Biomarkers

## Abstract

Multiple sclerosis (MS) is a demyelinating and neurodegenerative disease due to an autoimmune chronic inflammatory response, yet the etiology is currently not completely understood. It is already known that physical activity plays an essential role in improving quality of life, especially in neuropathological conditions. The study was aimed to investigate the possible benefits of high-intensity interval training (HIIT) in bone and lipid metabolism markers, and neuromotor abilities in MS patients. 130 participants were recruited; 16 subjects with MS met the inclusion criteria and were included in the data analysis. The patients were randomly assigned to two groups: a Control group (CG) (34.88 ± 4.45 yrs) that didn’t perform any physical activity and the Exercise group (EG) (36.20 ± 7.80 yrs) that performed HIIT protocol. The training program was conducted remotely by a kinesiologist. It was performed three times a week for 8 weeks. At the beginning (T0) and the end of the study (T1) physical function tests, bone remodelling markers, and lipid markers analyses were performed. After 8 weeks of training the wall squat (s) (T0 = 27.18  ±  4.21; T1 = 41.68 ± 5.38, *p* ≤ 0.01) and Time Up and Go test (s) (T0 = 7.65 ± 0.43; T1 = 6.34 ± 0.38 *p* ≤ 0.01) performances improved; lipid markers analysis showed a decrease in Total (mg/dl) (T0 = 187.22 ± 15.73; T1 = 173.44 ± 13.03, *p* ≤ 0.05) and LDL (mg/dl) (T0 = 108 ± 21.08; T1 = 95.02 ± 17.99, *p* < 0.05) cholesterol levels. Additionally, the levels of osteocalcin (µg/L), a marker of bone formation increased (T0 = 20.88 ± 4.22; T1 = 23.66 ± 6.24, *p* < 0.05), 25-OH Vitamin D (µg/L) improved after 8 weeks (T0 = 21.11 ± 7.11; T1 = 27.66 ± 7.59, *p* < 0.05). HIIT had an effect on lower limb strength and gait control, improved bone formation, and lipid management, in MS patients.

## Introduction

Multiple sclerosis (MS) is a chronic inflammatory demyelinating disease of the central nervous system. Its cause is currently complex and has an autoimmune background^1^. This condition mainly occurs in people aged 20 to 40 years old. The cause is not fully understood. However, two main factors, genetic and environmental, are known^2^.

Among the symptoms that characterize the disease, there are physical function disorders, such as walking and balance, systemic lipid metabolism alteration, and secondary osteoporosis. Previous studies showed a correlation between adverse clinical outcomes and increased serum cholesterol levels in MS^3^. Cholesterol levels have been indicated as biomarkers for disease progression^4^. In addition, pathologies characterized by motor dysfunction are related to changes in the balance of the bone resorption-formation process^5^.

The balance between the resorptive activity of osteoclasts and the production of new bone matrix by osteoblasts is known as bone remodeling^6^. It is controlled by mechanical stimuli and endocrine signals from calcium regulatory hormones such as parathyroid, calcitonin, and Vitamin D^7^. Bone mineral density (BMD) is affected in MS subjects^8^ who can often develop secondary osteoporosis^9,10^ due to reduced mechanical stimulation, deficiency in Vitamin D levels, and a secondary increase in intact parathyroid hormone (PTH)^8,11^ but also due to the use of certain drugs, e.g. during glucocorticoid therapy^12^. Exercise has a beneficial effect on metabolic processes. It can activate adaptive mechanisms based on the regulation of tissue plasticity processes, influencing the regulation of intracellular pathways^13^. Indeed, physical activity is a safe and recommended intervention for MS patients^14^, who have been shown to tolerate it^15^ and have benefits such as an increase in upper limb strength^16^. It also acts as a neuroprotective factor such as by increasing the brain-derived neurotrophic factor levels after 12 weeks of lactate threshold resistance exercise^1^. In addition, eight weeks of upper- and lower-body interval training using ergometers have been validated to help MS patients maintain normal blood–brain permeability^17^. Therefore, physical activity potentially plays an essential role in MS symptom management. However, there is no uniformity on what characteristics training protocols should have to be most suitable, safe, and effective for individuals with MS to achieve the predetermined goal. Various interventional studies have been published and there is an increase in interest in High-intensity interval training (HIIT) for MS^18^. It has been suggested that HIIT may be a beneficial treatment for MS patients, since it enables them to exercise more intensely without experiencing thermosensitive symptoms^19^ and in a shorter time. However, to our best knowledge, no one has tested the effect of this protocol on bone metabolism in MS participants.

It has already been shown that the most effective exercises in terms of load to stimulate the formation or maintenance of BMD are weight-bearing exercises, high-impact exercises (such as jumping), plyometric training, and weightlifting^20,21^. Aerobic training, such as cycling, walking, yoga, and swimming, although having a positive effect in maintaining homeostasis, seems not to have the same osteogenic effects. Moreover, long-duration endurance training, not supported by proper caloric intake, might have the opposite effect: promoting BMD decrease^22,23^. Therfore, we hypothesized that our HIIT protocol has good potential to be osteogenic because it involves resistance exercise and impact exercises with intermittent stimulus. In addition, several studies demonstrated the effect of HIIT on lipid profile management^24–26^. However, to date, there are conflicting studies on which protocol is most effective in managing the lipid profile^27,28^.

Different protocols are also able to improve motor function in neurodegenerative disease such as 11 weeks of resistance training improves motor function in subjects with Parkinson's disease^6^ and Lactate Threshold Training does the same in MS^1^. However, if HITT is compared with moderate continuous aerobic training and resistance training it induced the largest gains in physical function in healthy people^29^. Therefore, HIIT training has nearly the same potential effect as aerobic training on lipid profile management but it seems to have the largest effect on physical function and because of the characteristics of this stimulus it is potentially more functional for bone health.

Indeed, HIIT is typically achieved through the use of intervals^30^ and can be broadly defined as repeated bouts of exercise of short to moderate duration, which can range from 10 s to 5 min, completed at an intensity above the anaerobic threshold. Exercise bouts are interspersed with short periods of low-intensity work or inactivity that allow for partial but often incomplete recovery^30^. The goal of HIIT is to significantly increase the physiological systems that will be employed in comparison to what is necessary during the activity during a specific exercise of the endurance type^31^ to a significant extent compared to that required during the activity. Thus, the primary aim of our study is to investigate, for the first time, the effects of HIIT on bone remodeling. The second objective is to confirm or disprove the positive effects of a HIIT protocol on lipid management and physical function to understand the overall effectiveness of this protocol in symptom management of Relapsing–remitting MS patients with a minimal sign to Mild disability in a functional system.

## Materials and methods

### Study design

**Figure 1:**
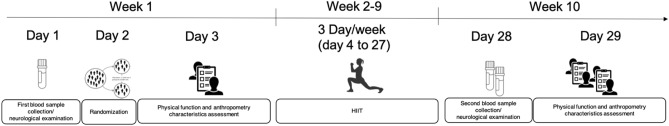
Study design.

This study was a randomized controlled trial. It was conducted according to the guidelines of the Declaration of Helsinki and approved by the Ethics Committee of The Palermo 1, decree N 7/2016 Policlinico Giaccone Hospital, Palermo, Italy. Written informed consent was obtained from participants before starting the study. Participants were recruited from the patient’s list of the Multiple Sclerosis Center of the Neurology Unit of the "Paolo Giaccone" University Hospital, which included a cohort of 130 subjects with a diagnosis of MS in observance of the McDonald's criteria revisions^32^. Only 30 patients from the patient’s list met the inclusion criteria. Of the 30 subjects who met the inclusion criteria, 16 subjects were available to participate in the study. The neurological and physical functions were performed on two different days and repeated at the end of the study after 8 weeks, always on two different days. After the first neurological analysis, the 16 participants were randomly assigned to two groups: 8 participants constituted the control group (CG) and 8 participants—the exercise group (EG). A Stratified Randomization^33^ with the sex of the participants considered as a stratification factor was used for randomization process. Breifly, the numbers indicating the order in the patient list by the 16 participants included represented the identifying number for each participant; they were written on separate pieces of paper and folded. The women's numbers (10) and the men's numbers (6) were placed inside two different boxes. After all participants have been assigned into blocks, simple randomization occurs: the first 5 numbers drawn from the women's group and the first 3 from the men's group would be part of the EG. The remaining numbers for both stratified groups would have been part of the CG. All participants performed the assessments before and after 8 weeks of the study. Figure 1 graphically describes the study design.

### Participants

The participants included in the study met the following inclusion criteria:

- Age range 20–55 years;
- Absence of clinical relapses in the 12 months preceding the study;
- Total Expanded Disability Status Scale (EDSS) score ranged between 1.5 and 3.5;
- Absence of concomitant diseases;
- MS diagnosed according to the McDonald criteria.

The participants were excluded from both the EG and the CG, if during the 8 weeks they had suffered relapses; if during the same period they had encountered comorbidities such as pathologies of the cardiovascular system, epilepsy, carcinomas, bone or muscle injuries; if they had changed their daily habits in diet or physical or work activity. Furthermore, to be included in the data analysis, the participants of the EG had to be present at more than 60% of the scheduled training sessions. An attendance register was filled in for the duration of the study. Regarding the clinical conditions, all patients had a relapsing–remitting MS with minimal to mild disability in a functional system. All subjects walked independently and were able to perform all proposed motor tests without the aid of any support. The EG performed the HIIT protocol during the 8-week training period and they had a mean score on the EDSS scale of 1.19 ± 0.59. The CG didn’t change their daily life habits (e.g. no scheduled exercise, no specific diet, etc.) during the same 8 weeks and they had a mean score on the EDSS scale of 1.75 ± 0.60. All participants didn’t perform any type of scheduled workout for at least one year before the study.

### Physical function and anthropometry characteristics assessments

Physical function and anthropometry assessments were performed at the Sport and Exercise Unit laboratory of the University of Palermo (via Giovanni Pascoli, 6, 90,144, Palermo, Italy) between 8 and 10 in the morning. The subjects were asked to fast for at least three hours to adequately detect their weight and standardize the caloric intake available to perform the tests. Before starting the Physical function assessment, personal information was requested, including age, and the weight was measured using the body composition analyzer “InBody_320_” (InBodyUSA, Cerritos, CA) scale and the height was measured using a wall-mounted stadiometer. All assessments were performed before the start of the exercise period protocol (T0) and at the end of the 8 weeks of training (T1). At of the T0 assessment, EG participants received a heart rate monitor (“Polar Vantage V2”; Polar Electro Oy, Kempele, Finland) to record the HR variation during the workout the . The EG participants were instructed how to record the data and were reminded to do it at the beginning of each workout. In addition, at the end of the physical function evaluation session, all subjects were showed the exercises of the three weekly sessions and emphasized any frequent errors.

Physical function was assessed through the following standardized tests:

- Timed up-and-go test (TUG): standardized test that allows to measure the level of functional mobility of the lower limbs and dynamic balance^34^. It has been shown that this test is indicative of neuromotor alterations as elderly subjects or those with neurodegenerative diseases appear to have scores below average^35^. The time in seconds (s) and tenths taken by the participant to get up from a chair without the aid of the upper limbs, walk 3 m, turn around, return to the chair, and sit down again was recorded with a digital stopwatch for each participant. After the explanation, the subject carried out a familiarization test immediately after the actual test during which the time was measured and used for the statistical analysis.
- Eye-hand reaction test: allows to evaluate reaction times as it records the time interval between the presentation of a visual stimulus and the execution of the response^36^. The participant was seated in a chair with the dominant limb positioned on the table in front where the computer was positioned. The arm and forearm had a 90° angle and the index finger rested on the response button located in the center of the computer. The subject had to press the button with the dominant limb once the light on the screen changed from red to green. The test involves the response to five stimuli, the software (https://faculty.washington.edu/chudler/java/redgreen.html) automatically shows the 5 results in s and calculates the average. The average value for each subject was taken into consideration for data analysis.
- Flamingo test: allows to measure the static monopodal balance. Participants were asked to stand on one leg, with the other leg flexed at knee height with the sole resting in the medial side of the knee of the weight-bearing leg. The arms should be at the sides. Participants must maintain the position for 60 s without shoes. The times the subject touches the ground with the non-weight-bearing foot are counted and the data used for statistical analysis is performed^37^. The test was performed one time on each side.
- Wall squat test: evaluates the isometric strength and functionality of the lower limbs. Participants had to maintain the squat position as long as possible, and time in s was recorded until the subject indicated the stop sign^38^**.** The standardized position was a 90° angle between leg and thigh, the back leaning against the wall. The test was performed once after proving the right position.
- Handgrip test: measures the maximum strength of the hand and forearm muscles using a digital dynamometer^39^. The subject sitting in a chair grasped the handle while maintaining a 90° angle between the arm and forearm. Then, the participant pushed the handle as hard as possible, maintaining the grip for 3 s. The test was repeated three times for each limb, one minute rest was taken between the three measurements of the same hand.^40^ The best result of the three tests for each limb was taken into account for statistical analysis. The force delivered was expressed in kg. Kern Map 80K1 (Kern® Kern & Sohn GmbH, Balingen, Germany) was used for the analysis.

### Training protocol

The patients from the EG exercised three times a week (Monday, Wednesday, and Friday), for 30 min per session for 8 weeks. An online live training conducted on the Google meeting platform was performed. An expert kinesiologist who managed the execution, the sequence, and the timing of the exercises was present at each training session and two of the co-authors (A.A. and A.A.) were supervising each session. On the first day of the training the exact camera position of each subject was ascertained by supervisors.

The protocol has been divided into three phases:

- Warm up;
- Work out;
- Cool down.

In the EG the warm-up and cool-down exercises (5 min each), were the same for all three weekly sessions and the participants performed joint mobility exercises (cervical, scapulohumeral, trunk, hip, knee, and ankle respectively) and stretching of the muscular structures in the aforementioned joint areas, in particular of the areas most involved in the training phase. The work out phase trained respectively the upper limbs (biceps and triceps brachii, deltoid, etc.), the lower limbs (quadriceps, hamstrings, gastrocnemius, soleus, etc.) and a combination of both for a total body workout in the three days. Overall, the sessions were carried out free-body with the help of small support (a chair, a table, or a bench), and the exercises were performed on a circuit where the intensity ranged from 65% HR during the active rest (30’’) to 85% during the exercise (30’’). The exercise intensity was calculated based on Age-Predicted Maximal Heart Rate^41–43^, collected with heart rate monitor previously described. Specifically, exercises performed during the central phase of the workout are illustrated in the table below (Table 1).

**Table 1:** Representation of the HIIT training protocol exercises performed during the 8 weeks.

First training session (upper limbs)	Second training session (lower limbs)	Third training session (total body)
Exercise (Set = 4; Reps = max in 30″)/Active rest (30’’)	Exercise (Set = 4; Reps = max in 30″)/Active rest (30’’)	Exercise (Set = 4; Reps = max in 30″)/Active rest (30’’)
Push-up/RP	Squat/RP	Push up/ST
Floor triceps/ST	Front lunges/ST	Squat/RP
Inclined push-up/RP	Intense march/RP	Crunch/ST
Bench dips/ST	Front lunges/ST	Floor triceps/RP
Mountain climber/RP	Crunch/RP	Jumping jack/RP

Reps = repetitions; RP = Run place; ST = Step touch; HIIT = high-intensity interval training.

### Bone and lipid markers analysis

The blood samples were taken in a fasting state two times at T0 and T1. Each blood sample was collected in special tubes to obtain serum. The tube did not contain an anticoagulant but contained a clot activator and serum separator gel.

Samples were centrifuged at 1300 × g for 15 min for fractionation of the samples at room temperature to obtain serum. The fresh serum was used to measure the following chemical markers using automated procedures according to standard commercially available assays supplied by Roche Diagnostics performed on the Roche COBAS c503. Triglycerides (TG), total cholesterol, cholesterol LDL, and cholesterol HDL were measured by colorimetric assay. As previously reported^44,45^, the samples were analyzed for the marker of bone resorption C-terminal telopeptide (β-CrossLaps), the marker of bone formation osteocalcin, the parathyroid hormone (PTH), and Vitamin D as markers of bone metabolism. Blood sample analysis was performed at the central laboratory analysis at the University of Palermo, Palermo, Italy.

### Statistical analysis

The normality distribution was verified using the Shapiro–Wilk test. Following the results of the normality check, to test the differences between T0 and T1 the Student’s t-test for paired data was performed. The results are expressed as mean and standard deviation. The p-value was considered significant for values < 0.05. JAMOVI Software (Version 2.3) was used for all statistical analyses (The Jamovi project (2022), retrieved from https://www.jamovi.org).

### Informed consent

The study was conducted according to the guidelines of the Declaration of Helsinki and approved by the Ethics Committee of The Palermo 1, decree N 7/2016 Policlinico Giaccone Hospital, Palermo, Italy.

## Results

The compliance for both the control and exercise groups was 100%. All sixteen participants took part in all phases of the study planned for each group and were included in the data analysis. The adherence of EG to the 8 weeks of training was 71.54% of scheduled sessions. The anthropometric characteristics of the subjects were the following: CG, age: 34.88 ± 4.45 years; height: 168.25 ± 8.66 cm; weight: 72.31 ± 17.28 kg and the EG, age: 36.20 ± 7.80 years; height: 170.40 ± 11.96 cm; weight: 72.70 ± 16.69 kg.

### Lipid profile markers

The hematological parameters assessment indicated a significant reduction of the lipid profile after 8 weeks of training in the EG. Particularly, the total cholesterol decreased from 187.22 ± 15.73 mg/dl to 173.44 ± 13.03 mg/dl, *p* < 0.045 and the LDL cholesterol level decreased from 108 ± 21.08 mg/dl to 95.02 ± 17.99 mg/dl, *p* < 0.022. No significant alterations were detected in the CG after 8 weeks. HDL cholesterol levels significantly decreased only in the CG (T0, 27.63 ± 7.50 mg/dl; T1, 18.13 ± 5.08 mg/dl), (Table 2).

**Table 2:** Lipid and bone marker changes in the CG and EG between T0 and T1.

Variables	CG			EG		
	T0	T1	p value	T0	T1	p value
	M ± SD	M ± SD		M ± SD	M ± SD	
Total CHOL (mg/dl)	166.62 ± 20.94	166 ± 27.36	0.897	187.22 ± 15.73	173.44 ± 13.03	0.045*
LDL (mg/dl)	80.62 ± 10.12	85.12 ± 16.67	0.443	108 ± 21.08	95.02 ± 17.99	0.022*
HDL (mg/dl)	27.63 ± 7.50	18.13 ± 5.08	< 0.001**	60.11 ± 18.31	56.33 ± 22.13	0.23
PTH (ng/L)	17.12 ± 9.94	4.52 ± 0.56	0.007**	39.77 ± 16.41	46.33 ± 15.10	0.176
Osteocalcin (µg/L)	17 ± 6.16	14.50 ± 5.12	0.017*	20.88 ± 4.22	23.66 ± 6.24	0.025*
25-OH Vitamin D (µg/L)	19.37 ± 6.32	21.25 ± 5.84	0.110	21.11 ± 7.11	27.66 ± 7.59	0.043*
β-CrossLaps (µg/L)	0.22 ± 0.08	0.29 ± 0.15	0.041*	0.35 ± 0.10	0.37 ± 0.20	0.690

***Statistically significant difference, *p* ≤ 0.001; **statistically significant difference, *p* ≤ 0.01; CG control group; EG exercise group; T0 = time before the start of the exercise period protocol; T1 = time at the end of the 8 weeks of training; M = mean; SD = standard deviation; CHOL = cholesterol; LDL = low-density lipoproteins; HDL = high density lipoproteins; PTH = parathormone.

### Bone metabolism and turnover markers

In the CG, there were significant changes in markers of bone remodeling after 8 weeks of study protocol compared to baseline. The concentrations of β-CrossLaps in CG increased (0.22 ± 0.08 *vs* 0.29 ± 0.15 µg/L, *p* < 0.041), while the levels remained unchanged in the EG. Concerning the bone formation markers osteocalcin, an opposite effect was found. Indeed, it decreased significantly in the CG (17 ± 6.16 *vs* 14.50 ± 5.12 µg/L, *p* < 0.017), while it increased in the EG (20.88 ± 4.22 *vs* 23.66 ± 6.24 µg/L, *p* < 0.025). As for PTH levels, they showed a significant reduction only in the CG (17.12 ± 9.94 *vs* 4.52 ± 0.56 ng/L, *p* < 0.007), while it remained unchanged in the EG. Significant change was found in vitamin D levels in the EG. The values varied from 21.11 ± 7.11 to 27.66 ± 7.59 µg/L, *p* < 0.043. Table 2 showed Lipid profile, bone metabolism, and turnover markers results as mean and standard deviation.

### Physical function assessment

The changes (mean and standard deviation) in the motor and coordination evaluation tests performed after the 8-week training period, are shown in Table 3. A significant improvement was observed in the EG for the timed up-and-go test (s) (T0 7.65 ± 0.43; T1 6.34 ± 0.38; p = 0.01) while the CG increased significantly the time needed to perform TUG after 8 weeks of the training (T0, 9.07 ± 0.55; T1, 10.96 ± 0.85; p = 0.003). In addition, EG also improved the performance of the wall squat test (s) (T0, 27.18 ± 4.21 *vs* T1, 41.68 ± 5.38, *p* < 0.002). There was no statistically significant change in the same test for the CG.

**Table 3:** Physical function assessments in CG and EG at T0 and T1 time points.

Variables	CG			EG		
	T0	T1	P value	T0	T1	P value
	M ± SD	M ± SD		M ± SD	M ± SD	
TUG (s)	9.07 ± 0.55	10.96 ± 0.85	0.003*	7.65 ± 0.43	6.34 ± 0.38	0.001**
Eye-Hand Reaction Test (s)	0.494 ± 0.49	0.491 ± 0.04	0.895	0.33 ± 0.01	0.38 ± 0.02	0.071
Flamingo test	5.75 ± 2.92	2.12 ± 1.46	0.132	4.88 ± 2.16	3.88 ± 1.65	0.438
Wall squat test (s)	18.47 ± 4.12	35.38 ± 8.04	0.054	27.18 ± 4.21	41.68 ± 5.38	0.002**
R Handgrip test (kg)	26.08 ± 3.66	26.23 ± 3.71	0.894	33.48 ± 2.52	33.96 ± 2.98	0.734
L Handgrip test (kg)	25.76 ± 2.83	24.80 ± 3.23	0.278	31.88 ± 2.37	32.67 ± 2.83	0.417

**Statistically significant difference, *p* ≤ 0.01 *statistically significant difference, *p* ≤ 0.05; CG control group; EG exercise group; T0 = time before the start of the exercise period protocol; T1 = time at the end of the 8 weeks of training; M = mean; SD = standard deviation; R = right; L = left.

## Discussion

HIIT is efficacious in improving fitness levels and is safe in people with MS^18^. However, so far, the efficacy of HIIT training in affecting metabolic parameters has not been investigated in this population. The present study showed that 8 weeks of HIIT increased the Osteocalcin (*p* < 0.05) and 25-OH Vitamin D (µg/L) (*p* < 0.05) levels and decreased the total CHOL (mg/dl) (*p* < 0.05) and LDL (mg/dl) (*p* < 0.05) levels in MS population. In addition, this training protocol improved the performance of the TUG (*p* < 0.01) and the wall squat (*p* < 0.01) tests.

Previous studies have demonstrated increased levels of osteocalcin and vitamin D3 in a healthy population following 8 weeks of HIIT, confirming our results^46^. However, the training protocol by Lu et al. involved healthy participants who performed only running on the treadmill (therefore no resistance exercise) during which the various intensities were interspersed. However, it is well known that to obtain an effective osteogenic stimulus, the succession of exercises must include resistance exercises directly involving all body segments. Resistance training results in an increase in site-specific bone density and is potentially the most effective type of exercise on bone mineral density (BMD) if performed for at least 3 sessions per week^47^ associated with “high impact/ground reaction” stimuli, another key factor in stimulating bone formation activities^48^. Thus, starting from the principles of the osteogenic potential of physical activity, to pursue our study’s aim, our protocol deviates from Lu's by showing that the same effects can be achieved with potentially more effective exercises for bone remodeling, taking the metabolic power of HIIT and in less time (45 min for one session^46^ versus 30 min total in our session). We have previously demonstrated the effect of exercise on the osteocalcin marker, but its increase was following classical resistance training (90 min per session) also in subjects with neurodegenerative diseases^6^. Demonstrating that HIIT could have a better or equal effect could be useful as one could achieve the same results but in a shorter time. Time is a key element in the daily management of subjects with MS but also of their caregivers. In addition, osteocalcin was significantly reduced in the sedentary group and these underlying osteocalcin level management are affected by the training period.

Vitamin D deficiency is characteristic of neurodegenerative diseases, including MS. Reduced Vitamin D levels are linked with bone resorption because it plays an essential role in calcium metabolism^49^. The neuroprotective role of vitamin D have already been demonstrated^50^. In our study, for the first time participants with MS starting from below-average vitamin D3 levels significantly increased their levels as a result of this exercise. In addition, β-CrossLaps, the serum marker of bone resorption, was increased and the PTH levels were significantly reduced only in the CG. The mechanism behind the reduction of PTH secretion in the sedentary group is unknown. However, consideringof the central role of PTH in regulating bone metabolism^51^, this result suggests that in the sedentary group, there is an altered PTH metabolism which negatively affects bone remodeling. These two results support our thesis about the effect of HIIT on bone metabolism.

One of the possible mechanisms that explains the effect of HIIT on bone metabolism is the production of lactate that occurs during this training. Indeed, it's shown that lactate mediates the bone anabolic effect of HIIT in osteoporosis, which results from enhanced osteoblast differentiation and mineralization^52^.

HIIT training in MS patients ameliorated strength, particularly referring to lower limb function. The wall squat test assesses the isometric strength of the lower limbs giving information relevant to the functionality of the muscles of the hips and thighs, key muscle districts in balance maintenance strategies^53^. Our results are in agreement with past studies that have already demonstrated the effect of HIIT training on lower limb strength^54,55^. Zaenker et al. showed that 12 weeks of HIIT improved strength in MS. However, they performed an isokinetic test to analyze lower limb strength improvement but for our study’s aim, we chose to evaluate the strength improvement with a standardized functionality test for this population.

The TUG has highly reliable gait and balance tests in individuals with MS^56,57^ it gives the important information on static and dynamic balance control during walking ability often impaired in individuals with neurodegenerative diseases^58,59^. Manca et al. showed that 6 weeks of HIIT improved TUG performance in MS patients^54^. However, their training involved just the lower limb stimulation, and the exercises were performed under a load. The simultaneous improvement in walking performance and wall squat test may be attributed to the intervention because of the increased strength of stabilizing muscles such as those of the hip and thigh influence.

It is known that lipid metabolic pathways are crucial in MS patients for the process of remyelination. Elevated LDL serum levels were correlated with negative progression of the disease^60,61^. HIIT training by reducing total cholesterol and LDL may act in slowing disease progression by preserving lipid metabolism.

While the exact mechanisms underlying the effect of exercise on the lipoprotein profile are unclear, it is known that exercise improves the ability of skeletal muscles to utilize lipids^62^. HIIT initially causes a decrease in adenosine triphosphate (ATP) reserves followed by a decrease in glycogen reserves through the anaerobic glycolysis^63^. HIIT is characterized by an intensity of the workout above the anaerobic threshold and the variations between intensities inducing adaptation of metabolic processes, such as aerobic capacity^64^ and insulin sensitivity^65^ of exercised muscle groups that can improve fat metabolism.

Following our results, Van Ryckeghem et al. Showed an improvement in lipid profile, particularly in total cholesterol levels, following 24 weeks of HIIT in subjects with type 2 diabetes mellitus^25^. They used a protocol similar to ours in intensity and session duration performing 1-min high-intensity exercise at 85% VO_2_peak with a 4-min low-intensity exercise at 60% VO_2_peak, 30 min total each session. Moreover, even in this study, HDL levels did not change thus assuming a direct effect of HIIT selectively on “bad” cholesterol.

Similar results were achieved by Mitranun et al. who showed a decrease in total cholesterol (*p* < 0.05) and LDL (*p* < 0.05) after 12 weeks of HIIT and showed that this variation was only in the HIIT group when compared with a sedentary group and continuous training^26^.

The results were also confirmed by a systematic review affirming the effectiveness of HIIT, compared with other workouts, in managing lipid profiles in this population^66^.

Instead, Jorissen et al. contrast our study, demonstrating that comparing HIIT training to medium-intensity continuous training, the latter has a greater effect on the lipid profile of MS participants^67^. The authors justified the result with the greater work volume of medium-intensity continuous training which seemed to influence the lipid profile in more extend. However, there are conflicting studies on the effect of other types of training on lipid profile. For example, while Mandrup et al. demonstrated that continuous high-intensity training-induced reduction in LDL-C, total cholesterol, and total cholesterol/HDL-C index^24^, the STRRIDE^68^ and the American Heart Association^69^ states that there is no effect of moderate or vigorous continuous exercise on LDL-C6 concentrations. This indicates that, while there seem to be differing opinions on the effects of various exercise modalities on lipid profile, HIIT seems to have more homogeneous results and thus should be the recommended protocol for the best management of lipid profile.

In addition, EG participants in our study fluctuated between 3 and 4 heart rate zones^70^ during the training that, in the ACSM guidelines, the intensity classification of physical activity corresponds to a range between moderate (64% to 76% of maximal heart rate) and vigorous (85% and above of maximal heart rate)^71^, being adequate to cause the effects just described. High-intensity interval training guidelines include a variation between high-intensity and light-moderate intervals, where recovery periods can last as long as work periods for a total per session that should be between 20–60 min^72,73^.It is important to underline that multiple aspects beyond training such as possible underlying inflammatory processes in MS patients could easily influence their lipoprotein profile.

## Conclusion

In conclusion, no adverse events occurred during the training. HIIT training confirmed to be an effective and safe training method for subjects with multiple sclerosis. In addition, the study showed for the first time the effectiveness of HIIT on bone remodelling, lipid profile, and functionality of lower limbs in MS patients and suggests that HIIT training could be used to preserve bone status and slow the occurrence of osteoporosis. It also seems to be a good tool for maintaining a healthy lipid metabolism.

### Limitations and future perspectives

This study has several limitations. First, stratified randomization created an unequal distribution of baseline values in the two groups. In addition, online live training was conducted. While the effectiveness of the training conducted online on healthy adults during the COVID-19 pandemic state was experinced^74,75^ and this might be a positive aspect of time and space management, this method affected the socialization of the group and the ability of the kinesiologists to catch any errors in the execution of the exercises due to interruptions in the internet connection or poor framing. Moreover, considering the relevance of these preliminary results, additional studies are needed with larger sample sizes and longer intervention periods. In addition, since we do not plan for follow-up, we do not know the permanence of these effects. This could be a direction for future studies.
